# “Carry-Over” Effect of CDK4/6 Inhibitors in Adjuvant Therapy for Hormone Receptor (HR)-Positive/HER2-Negative Early Breast Cancer: Clinical Evidence and Molecular Approach

**DOI:** 10.3390/biomedicines14040893

**Published:** 2026-04-14

**Authors:** Guillermo Valencia, Zaida Morante, Yomali Ferreyra, Rosario Jacome, Patricia Rioja, Alexandra Saavedra, Silvia Neciosup, Tatiana Vidaurre, Henry L. Gómez

**Affiliations:** 1 Oncosalud—AUNA, Lima 15036, Peru; zmorante@gmail.com (Z.M.); patyriojavi@gmail.com (P.R.); silvianeciosup@yahoo.com (S.N.); hgomezmoreno@gmail.com (H.L.G.); 2 Medical Oncology Department, Instituto Nacional de Enfermedades Neoplásicas (INEN), Lima 15038, Peru; ale6936macarena@gmail.com (A.S.); tatiana.vidaurre@gmail.com (T.V.); 3 Faculty of Medicine, Universidad San Ignacio de Loyola (USIL), Lima 15024, Peru; 4 Health Innovation Laboratory, Institute of Tropical Medicine “Alexander von Humboldt”, Universidad Peruana Cayetano Heredia (UPCH), Lima 15102, Peru; yomaliferreyra17@gmail.com; 5 School of Engineering, Universidad de Ingeniería y Tecnología (UTEC), Lima 15063, Peru; rosario.jacome@utec.edu.pe

**Keywords:** early breast cancer, hormone receptor (HR)-positive/HER2-negative, CDK4/6 inhibitors, adjuvant therapy, “carry-over” effect

## Abstract

**Background**: Hormone receptor-positive/HER2-negative (HR+/HER2−) early breast cancer (EBC) presents a persistent risk of relapse, even beyond 5 years, driving the need for adjuvant intensification strategies. This review analyzes the clinical evidence for CDK4/6 inhibitors (CDK4/6i) in the adjuvant setting. This evidence is then integrated with molecular findings to support the concept of the “carry-over” effect, which is understood as a lasting benefit that persists after the end of active treatment, reflected by a sustained separation of invasive disease-free survival (iDFS) curves during follow-up. **Relevant Sections**: The main adjuvant trials in EBC are reviewed, with consideration of the “carry-over” effect. Emerging biomarkers and the impact of financial toxicity are also described. **Results**: PALLAS did not demonstrate a clear on-treatment or post-treatment benefit, whereas PENELOPE-B suggested, at most, a transient early advantage that was not maintained with longer follow-up; therefore, neither trial provides convincing evidence of a durable “carry-over” effect. In contrast, monarchE (abemaciclib) and NATALEE (ribociclib) showed significant improvements in iDFS and, in the case of abemaciclib, a signal of benefit in overall survival, supporting the existence of a clinically relevant post-treatment effect. **Conclusions**: From a biological perspective, the review proposes that the “carry-over” effect should not be considered a uniform class effect, but rather the result of a sequence of events modulated by pharmacological selectivity (CDK4 vs. CDK6 and additional targets), the induction of cellular senescence, and immunomodulatory effects that could favor the control of micrometastases. In addition, elements that influence interpretation and the need to optimize adherence and toxicity management to “materialize” the benefit in a potentially curable context are discussed.

## 1. Introduction

Luminal early breast cancer (HR+/HER2− EBC) accounts for approximately 70% of all breast cancer cases. It is characterized by an indolent course and a persistent risk of late recurrence, even beyond 5 years after initial treatment [1]. Notably, up to 30% of patients experience recurrence, a rate significantly higher than other subtypes of breast cancer, underscoring the importance of targeted treatment strategies. Most patients are diagnosed at an early stage, and the staging (tumor size and nodal involvement) is a strong predictor of recurrence [2].

Major risk factors include family history, older age, prolonged estrogen exposure, and obesity. In patients with HR+/HER2− EBC, these factors are compounded by the possibility of late relapses despite patients receiving standard therapies [3]. The sustained risk of recurrence has driven the study of more effective, targeted therapies such as cyclin-dependent kinases (CDK) 4/6 inhibitors, which, when combined with endocrine therapy (ET), aim to improve invasive disease-free survival (iDFS), a key endpoint associated with delayed metastatic relapse and disease progression [4].

Although CDK4/6 inhibitors, such as abemaciclib and ribociclib, have been approved for adjuvant treatment of HR+/HER2− EBC, questions remain regarding the optimal ET partner selection, toxicity management, treatment exposure, and interpretation of durable benefit [5]. Likewise, replacement strategies with innovative therapies are being evaluated [6,7].

The concept of a “carry-over” effect refers to the persistence of anticancer benefit after completion of a finite course of therapy; this idea is well established in adjuvant endocrine therapy and is especially relevant in HR+/HER2− EBC, where recurrence risk extends over many years.

In this review, we define the “carry-over effect” as the persistence of clinical benefit after completion of a finite adjuvant CDK4/6 inhibitor therapy. Clinically, this is demonstrated by the maintained or increasing separation of invasive disease-free survival (iDFS)/distant recurrence-free survival (DRFS) curves beyond treatment discontinuation, interpreted together with hazard ratios, landmark analyses, and follow-up duration; biologically, it may reflect durable control of micrometastatic disease through senescence-associated and immune-mediated mechanisms.

## 2. Phase III Adjuvant Clinical Trial Data

To date, various trials with CDK4/6 inhibitors as adjuvant therapy have not demonstrated a “carry-over” effect (PALLAS and PENELOPE), whereas others have (monarchE and NATALEE), as shown in Table 1.

(a) PALLAS trial

This phase III, open-label PALLAS clinical trial evaluated the efficacy of adding palbociclib to adjuvant ET for 2 years in patients with HR+/HER2− stage II–III EBC (n = 5753). No statistically significant improvement in iDFS was achieved: the 3-year iDFS rate was 88.2% with palbociclib + ET vs. 88.5% with ET alone (HR: 0.93; 95% CI, 0.76–1.15; *p* = 0.51) [8]. One of the main factors that may have influenced the results was the high overall discontinuation rate of palbociclib (42.4%), with 21.7% of patients discontinuing due to adverse events, mainly neutropenia (83.5%) and fatigue (41.0%) [8,9]. Even though 58.7% of patients had “high-risk” disease, the broader-risk population in PALLAS might have diluted the detectable magnitude of benefit compared with trials enriched for higher-risk disease. In contrast, abemaciclib has more effective toxicity management strategies, which include proactive dose adjustments and early intervention for side effects such as diarrhea. Toxicity management strategies, regularly scheduled monitoring, and timely dose adjustments can significantly reduce AEs. Early intervention for common side effects can also be crucial. For instance, patients experiencing diarrhea can benefit from immediate anti-diarrheal medications, and those with neutropenia may require growth factor support or temporary treatment pauses to maintain optimal safety and adherence. It positions toxicity management as part of the “carry-over equation” and sharpens clinical relevance by suggesting actionable practice changes to reduce discontinuation rates.

Although subgroup analyses did not show an overall clinical benefit, a subsequent translational analysis identified a composite biomarker (luminal A with ERBB2-related signaling features and/or ER-positive/PgR-negative) associated with a significant improvement in iDFS with palbociclib (HR: 0.55; 95% CI, 0.34–0.90; *p* = 0.017).

Long-term follow-up analysis of the PALLAS trial showed no overall survival (OS) benefit with palbociclib + ET compared with ET alone (5-year OS was 92.6% with palbociclib vs. 93.2% with ET alone, HR 1.09, 95% CI 0.89–1.33, *p* = 0.4051). Moreover, no significant iDFS benefit of palbociclib was observed (5-year iDFS: 84.2% vs. 82.4%, HR 0.88) across all subgroups evaluated [9]. Exploratory analyses from PALLAS showed no benefit regardless of clinical stage (IIA vs. IIB/III) [10] or with concomitant medications such as proton pump inhibitors (PPIs); their use was not associated with higher rates of neutropenia [11].

(b) PENELOPE-B trial

The PENELOPE-B trial was a phase III, randomized, double-blind, placebo-controlled clinical study that evaluated the efficacy of palbociclib as an adjuvant treatment in women with HR+/HER2− EBC with residual disease after neoadjuvant chemotherapy (n = 1250). Eligible patients had a “high risk” of relapse, defined by a CPS-EG score ≥ 3 or 2 with positive nodal involvement at surgery (ypN+). Patients were assigned to receive palbociclib (125 mg orally for 21 days of a 28-day cycle, for 1 year) plus standard ET or ET plus placebo. The primary endpoint was iDFS, while secondary endpoints included distant recurrence-free survival (DRFS), OS, pharmacokinetics (PK), safety, and quality of life.

After a median follow-up of 42.8 months, the study did not meet its primary endpoint, with a 3-year iDFS rate of 81.2% in the palbociclib group vs. 77.7% in the placebo group (HR: 0.93; 95% CI: 0.74–1.17; *p* = 0.525). No significant differences were observed in OS (HR: 0.87; 95% CI: 0.61–1.22; *p* = 0.420) or DRFS. The most frequent AEs with palbociclib were neutropenia (95.7%), leukopenia (99.2%), and anemia (73.9%), with grade 3–4 events in 70.0%, 56.1%, and 3.2%, respectively. The treatment discontinuation rate was 19.6%, with 5.3% attributed to toxicity. PENELOPE-B failed to demonstrate an iDFS improvement in patients with residual disease after neoadjuvant treatment. Despite a selected high-risk population and good treatment adherence, the overall results of PENELOPE-B did not support the routine use of palbociclib [12].

A final survival report from the PENELOPE-B trial showed no significant improvement in iDFS (6-year iDFS 65.1% vs. 64.5%, HR 0.94), DRFS, OS (6-year OS: 82.4% vs. 80.3%, HR 0.87), or locoregional relapse rate. No benefits across major subgroups were found, except in lobular breast cancer (LBC), with a trend toward better survival outcomes in favor of palbociclib (HR 0.45, 95% CI 0.19–1.07, *p* = 0.062 for OS and HR 0.52, 95% CI 0.28–0.97, *p* = 0.035 for iDFS) [13].

**Figure 1:**
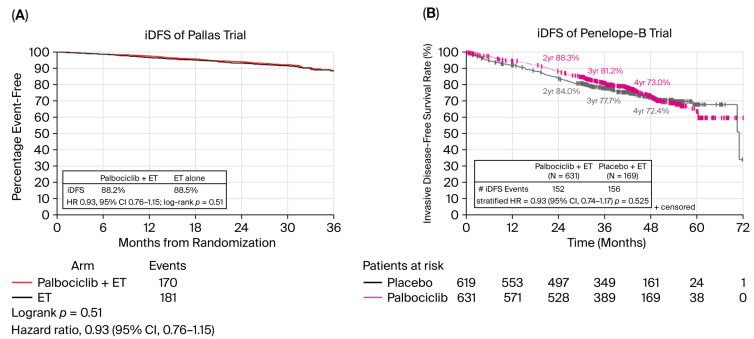
iDFS Kaplan–Meier plots based on results from the negative palbociclib-based adjuvant trials PALLAS (**A**) and PENELOPE-B (**B**) at a median follow-up of 36 and 42.8 months, respectively.

Figure 1 shows the Kaplan–Meier curves for iDFS in the PALLAS and PENELOPE-B trials.

In an exploratory analysis of the PALLAS and PENELOPE-B trials, an iDFS benefit was observed only in patients with a favorable genomic profile, which included the luminal A subtype and low levels of the serum marker thymidine kinase 1 (TKa) at baseline and after 7 months of treatment (HR 0.63; 95% CI 0.42–0.95; *p* = 0.025) compared to those without these biomarkers (HR: 1.11; *p* = 0.56) [14]. Currently, these biomarkers are under investigation for clinical application. While not yet standard, they have the potential to enhance patient selection by identifying individuals who may benefit most from CDK4/6 inhibitor therapies.

(c) monarchE trial

The phase III monarchE study evaluated the efficacy of abemaciclib in combination with adjuvant ET in patients with HR+/HER2− EBC (n = 5637) with a “high risk” of recurrence, defined as ≥4 positive lymph nodes (N2) or 1–3 nodes with high-risk features (tumor ≥ 5 cm, histologic grade 3, or Ki-67 ≥ 20%). Patients received abemaciclib 150 mg twice daily (BID) for 24 months. At 3 years of follow-up, the abemaciclib + ET group showed a significant improvement in iDFS, with a 3-year iDFS rate of 89.1% compared to 83.4% with ET alone, reflecting an absolute difference (Δ) of 5.7%. At 5 years, the rates were 86.7% vs. 80.6% with ET alone (HR 0.68; 95% CI 0.599–0.772; *p* < 0.001; Δ 6.5%). Distant metastasis-free survival (DMFS) also improved significantly (HR 0.68; 95% CI 0.565–0.820; *p* < 0.001) with abemaciclib + ET. Regarding safety, common AEs such as diarrhea (83%), neutropenia (45.2%), and fatigue (39.2%) were reported and were generally manageable with dose adjustments [15].

The efficacy of adjuvant abemaciclib was maintained across subgroups, including older patients (≥65 years), although with a higher incidence of gastrointestinal AEs and treatment discontinuations [16].

**Table 1:** CDK4/6 inhibitor phase III trials in HR-positive/HER2-negative early breast cancer.

Trial	Treatment	Population (n)	iDFS Rate (%)	CDK4/6 Inhibitor Duration	Landmark Efficacy Results and Maturity of Follow-Up	Toxicity (Common AEs)	Key Messages
PALLAS [8,9,10,11]	Palbociclib + ET	“High-risk” HR+/HER2− EBC (n = 5753)	88.2	2 years	3 years No “carry-over” effect	Neutropenia (83.5%)	No iDFS benefit. High discontinuation rate (42.4%). Discontinuation due to AEs: 21.7%. The subgroup with a composite biomarker (luminal A, low levels of TKa) showed a benefit.
PENELOPE-B [12,13]	Palbociclib + ET	HR+/HER2− EBC (n = 1250)	81.2	1 year	42.8 months No “carry-over” effect	Neutropenia (95.7%)	No overall iDFS improvement. Treatment discontinuation rate of 19.6%, of which 5.3% was due to toxicity. Benefit only in a subgroup with a favorable genomic profile.
monarchE [15,16,17,18,19]	Abemaciclib + ET	High-risk stage II–III HR+/HER2− EBC (n = 5637)	86.7	2 years	5-year: iDFS: Δ 6.5% DRFS: Δ 5.1% OS: Δ 1.8% “Carry-over” effect observed	Diarrhea (83%), neutropenia (45.2%), fatigue (39.2%)	Significant improvement in iDFS, DRFS, and OS. Discontinuation due to AEs: 18.5%. Effective in high-risk patients.
NATALEE [20,21,22,23]	Ribociclib + ET	Stage II–III HR+/HER2− EBC, including N0 (n = 5101)	90.4	3 years	3-year: iDFS Δ 3.3% 4-year: iDFS Δ 4.9% 5-year: iDFS Δ ~4.5% “Carry-over” effect observed	Neutropenia (44% grades 3–4), elevated liver enzymes, QTc prolongation	Benefit in intermediate- and high-risk disease. Includes a broad patient population (stage II, node-negative). Discontinuation due to AEs: 19%.

An update at 6.3 years of follow-up from monarchE continued to demonstrate sustained benefit in iDFS (7-year iDFS: 77.4% with abemaciclib + ET vs. 70.9% with ET), DRFS (7-year DRFS 80.0% with abemaciclib + ET vs. 74.9% with ET alone), and OS (7-year OS: 86.8% with abemaciclib + ET vs. 85.0% with ET alone, Δ 1.8%). The OS benefit was consistent across all evaluated subgroups, including fewer patients with distant metastases in the abemaciclib group. These results support the use of abemaciclib as adjuvant therapy in “high-risk” patients, being the first CDK4/6 inhibitor to demonstrate an emerging OS benefit in the adjuvant setting in HR+/HER2− EBC [17]. These outcomes led to the approval of abemaciclib by international health authorities for the treatment of HR+/HER2− EBC patients with node-positive disease at high risk for recurrence [18,19].

**Figure 2:**
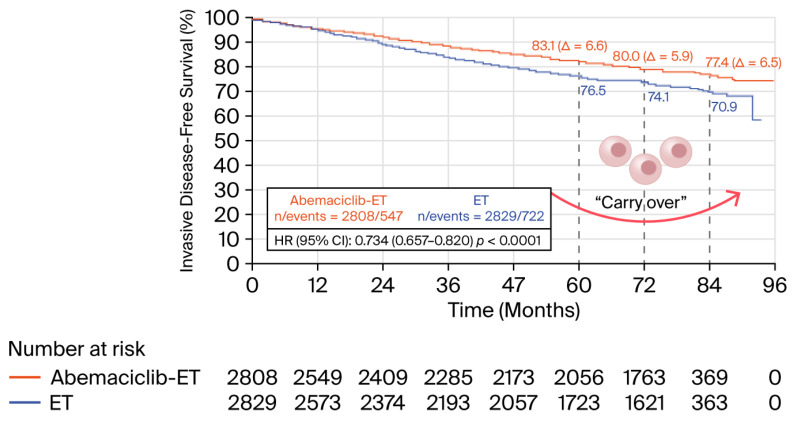
Long-term “carry-over” effect on 7-year iDFS as observed in the monarchE trial (2 years of treatment with abemaciclib).

One important consideration is that the absolute benefit of abemaciclib over ET alone, in terms of iDFS (Δ 6.5%), DRFS (Δ 5.1%), and OS (Δ 1.8%), is maintained throughout follow-up, supporting the hypothesis of a potential “carry-over” effect associated with this CDK4/6 inhibitor. These percentages have a tangible impact on patient care, potentially reducing the risk of disease recurrence and prolonging survival. During patient counseling or shared decision-making between clinicians and patients, these figures support the potential value of incorporating abemaciclib into adjuvant therapy. This data-driven dialogue can empower patients to make informed choices about their treatment options, balancing efficacy with life-quality considerations. As illustrated in Figure 2, the sustained plateau in iDFS over time visually supports the assertion of a “carry-over” effect, suggesting a long-term benefit beyond active treatment.

(d) NATALEE trial

The phase III, randomized, open-label NATALEE clinical trial evaluated the efficacy of ribociclib combined with ET, specifically a nonsteroidal aromatase inhibitor (NSAI), as an adjuvant treatment in patients with stage II and III HR+/HER2− EBC (n = 5101). Patients received ribociclib at a dose of 400 mg daily (3 weeks on treatment followed by 1 week off) for 36 months, in combination with an NSAI administered for at least 60 months. At 3 years, iDFS was significantly improved with ribociclib plus NSAI compared with NSAI alone (3-year iDFS: 90.7% vs. 87.6%; HR for invasive disease, recurrence, or death, 0.75; 95% CI 0.62–0.91; *p* = 0.0012), representing Δ 3.3%. This effect was maintained across evaluated subgroups, including node-negative patients (N0), those with stage II–III disease, and premenopausal patients. The most frequent AEs with ribociclib were neutropenia (44% grades 3–4), elevated liver enzymes, and corrected QT interval (QTc) prolongation, which occurred less frequently with the dose used than with the metastatic setting (600 mg). Almost 20% of patients discontinued ribociclib due to toxicity, despite the reduced dose. NATALEE included a broad patient population, expanding its clinical applicability [20].

**Figure 3:**
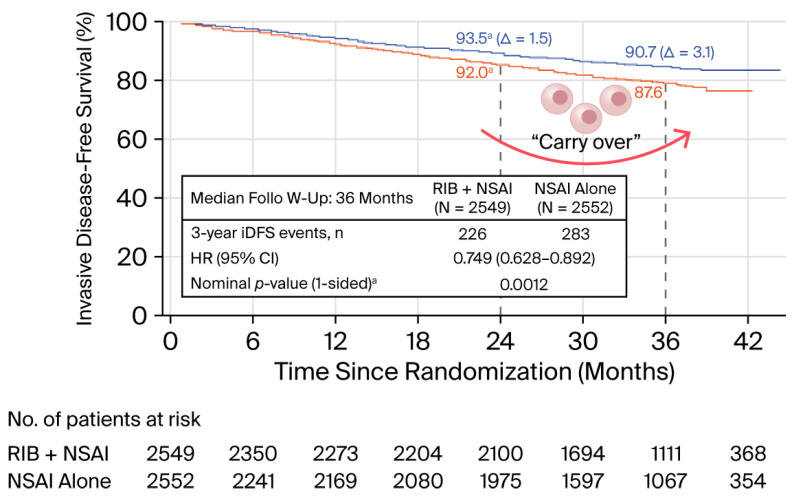
“Carry-over” effect of 36-month treatment period in NATALEE trial on iDFS (3 years of treatment with ribociclib). The blue line represents patients treated with adjuvant ribociclib, while the red line represents patients who received a placebo.

As observed after the 24-month treatment period in the monarchE study, a “carry-over” effect of ribociclib on iDFS after the 36-month adjuvant treatment period was observed during follow-up (Figure 3).

During 4 years of follow-up, iDFS was 88.5% with ribociclib + ET vs. 83.6% with ET alone (HR 0.72; *p* < 0.0001), Δ 4.9% [21]. A 5-year follow-up update showed that ribociclib + ET continues to provide a benefit vs. ET alone in HR+/HER2− EBC (Δ 4.5%) in iDFS and across all subgroups, including N0 disease (HR 0.60). The OS follow-up is ongoing, with a trend toward improvement in favor of ribociclib (HR 0.80, 0.637–1.003, *p* = 0.026) [22]. The NATALEE results had also led to regulatory approval of ribociclib in a broad group of patients [23].

## 3. Molecular Mechanisms and Clinical Interpretation of “Carry-Over” Effect

(a) Selectivity of CDK4/6 inhibitor

CDK4/6is are orally administered small molecules that competitively bind to the adenosine triphosphate (ATP)-binding cleft of CDK4 and CDK6 [24]. Although they all share the same primary mechanism of action, they exhibit distinct biochemical properties that influence their selectivity. Palbociclib and ribociclib are structurally similar, derived from a pyrido [2,3-d]pyrimidin-7-one scaffold [25], which features a large lipophilic chain. In contrast, abemaciclib is derived from a 2-anilino-2,4-pyrimidine-[5-benzimidazole] scaffold [24]. Structural differences could lead to variations in binding affinity and kinase selectivity; specifically, differentiation in CDK4 versus CDK6 affinity has been correlated with divergent survival outcomes in previous trials. Palbociclib shows relatively balanced inhibitory activity against CDK4 (Half-maximal Inhibitory Concentration [IC_50_] ~ 11 nanomolar [nM]) and CDK6 (IC_50_ ~ 15 nM), while ribociclib (CDK4 IC_50_ ~ 10 nM, CDK6 IC_50_ ~ 39 nM) and abemaciclib (CDK4 IC_50_ ~ 2 nM, CDK6 IC_50_ ~ 9.9 nM) display greater selectivity for CDK4. Notably, abemaciclib also targets other CDK family members (CDK1, CDK2, CDK5, CDK9, CDK14, CDKs16–18, GSK3α/β, CAMKIIγ/δ, and PIM1 kinases) [26]. This could contribute to a “carry-over” effect by inducing cellular senescence during follow-up and may, therefore, not represent a true “class effect” shared by all CDK4/6 inhibitors [27].

These pharmacologic and biologic features may contribute to the durability of the post-treatment benefit observed with abemaciclib, but at present, they should be considered mechanistic hypotheses rather than definitive explanations.

A key question for future trials is the optimal duration of ribociclib treatment: is there a greater benefit with a 36-month course than with a 24-month one? Currently, the recommended duration for ribociclib in adjuvant therapy is 36 months. This question could guide the design of next-step trials and help refine treatment protocols by determining whether extending the duration further enhances long-term outcomes.

Given the available evidence, it is not possible to fully disentangle drug-specific biology from treatment exposure, discontinuation, and trial-design factors as contributors to the divergent efficacy observed across adjuvant CDK4/6 trials. Rather than indicating a strict drug-specific dichotomy, the divergent adjuvant trial results likely reflect an interplay between pharmacology, trial design, treatment exposure, and disease-risk enrichment; notably, exploratory biomarker analyses in palbociclib trials suggest that selected biological subgroups may still derive benefit from CDK4/6 inhibition.

Cross-trial comparisons among PALLAS, PENELOPE-B, monarchE, and NATALEE should be interpreted with caution. These studies differed substantially in patient population, recurrence-risk enrichment, prior therapy exposure, treatment duration, ET backbone, discontinuation rates, toxicity profiles, duration of follow-up, and maturity of efficacy endpoints, particularly overall OS. Therefore, apparent differences in the magnitude or durability of benefit across trials cannot be attributed solely to drug-specific properties and should be considered exploratory rather than comparative evidence.

To provide a more comprehensive view of CDK4/6 inhibition in EBC, representative phase II studies such as PALLET, NeoPAL, and LEADER trials are also briefly discussed, which collectively show clear biologic activity and inform issues related to schedule, patient selection, and translational endpoints, even though they do not by themselves establish durable adjuvant benefit:

- PALLET: In this phase II randomized trial, the addition of palbociclib to letrozole in the neoadjuvant setting (n = 307) resulted in a more pronounced antiproliferative effect, as measured by a reduction in Ki-67 (−4.1 vs. −2.2, *p* < 0.001) and a higher rate of complete cell cycle arrest (90% vs. 59%, *p* < 0.001), but this did not translate into a clear increase in clinical or pathological response (*p* = 0.20; complete response + partial response, 54.3% vs. 49.5%) in the short term. In addition, grade ≥3 toxicity was more common (49.8% vs. 17.0%, *p* < 0.001), primarily due to neutropenia. These findings suggest robust biological activity, although not necessarily accompanied by greater early tumor reduction [28].
- NeoPAL: In this multicenter, phase II trial, the neoadjuvant combination of letrozole and palbociclib demonstrated clinical (iDFS: HR 0.83, 0.31–2.23, *p* = 0.71) and pathological activity (3.8% vs. 5.9%) comparable to that of neoadjuvant chemotherapy in patients with “high-risk” luminal breast cancer (n = 106), and in subsequent follow-up, survival outcomes were described as similar (progression-free survival: HR 0.83, 0.31–2.23, *p* = 0.71), suggesting that, in selected subgroups, this strategy could represent an alternative to avoid chemotherapy without compromising mid-term outcomes. However, given its sample size and phase II design, these results should be interpreted as hypothesis-generating rather than definitive evidence of lasting adjuvant benefit [29].
- LEADER: This prospective phase II trial explored the adjuvant use of ribociclib with two dosing strategies (400 mg continuous vs. 600 mg intermittent) and timing of initiation [early (prior endocrine therapy < 2 years, delayed (prior endocrine therapy ≥ 2 years)] in stage I-III HR-positive/HER2-negative EBC (n = 81). The trial showed that ribociclib was feasible and reasonably well tolerated, with no significant differences in serious adverse events between regimens; the trial also suggested that continuous administration at 400 mg and a later start time (ribociclib discontinuation was higher in early vs. delayed initiation, with discontinuation rates of 36% vs. 21%) might be associated with better tolerability. At a median follow-up of 20 months, recurrence was rare (2-year recurrence-free survival: 97% in the intermittent arm vs. 100% with the continuous arm), and a low ctDNA detection (only in two patients with recurrent disease) preceded radiological relapses, providing an interesting insight into the monitoring of minimal residual disease [30].

One possible biologic explanation for the differential durability of benefit is that greater CDK4 selectivity may favor more sustained cell cycle arrest and senescence-associated programs, a hypothesis further supported by the development of next-generation CDK4-selective inhibitors such as atirmociclib.

(b) Induction of cellular senescence

CDK4/6 inhibitors lead to a cytostatic arrest of tumor cells in the G1 phase by maintaining Retinoblastoma Proteins (pRBs) in an unphosphorylated state. A key question is whether this arrest is transient (reversible quiescence) or will become permanent (senescence). Studies have observed events such as the redistribution of the alpha-thalassemia mental retardation X-linked (ATRX) gene on chromatin and the sustained repression of proliferation promoters (e.g., HRAS, MYC) necessary to lock in the senescent state [31]. Senescent cells often develop a Senescence-Associated Secretory Phenotype (SASP), characterized by the secretion of cytokines, chemokines, and proteases. For instance, palbociclib has been shown to induce the overexpression of VCAM-1 and other pro-inflammatory SASP molecules in endothelial cells. Due to its additional inhibition of CDK9 and CDK2, abemaciclib produces unique transcriptional changes. CDK9, part of the Positive Transcription Elongation Factor b (P-TEFb) complex necessary for transcriptional elongation, when inhibited, can lead to a marked reduction in the expression of short-lived genes such as MYC and Myeloid Cell Leukemia-1 (MCL1) [32].

In fact, abemaciclib has been observed to reduce MYC levels more significantly than other inhibitors and rapidly sensitize MCL1-dependent cells to apoptosis. Additionally, abemaciclib may inhibit Glycogen Synthase Kinase 3 Beta (GSK3β), thereby activating the Wnt/β-catenin pathway and stabilizing nuclear β-catenin. Although Wnt/β-catenin typically promotes proliferation, in the presence of CDK4/6 blockade, the resulting cyclin D accumulation cannot drive the cycle forward and may sequester CDK inhibitors, thus altering feedback loops [33]. Palbociclib and ribociclib do not significantly impact the Wnt/GSK3β pathway [34]. A trial that described the dose–response curves showed that abemaciclib was 5.5 times more potent at inducing cytostasis than palbociclib based on growth rate 50 (GR50) values [35].

**Figure 4:**
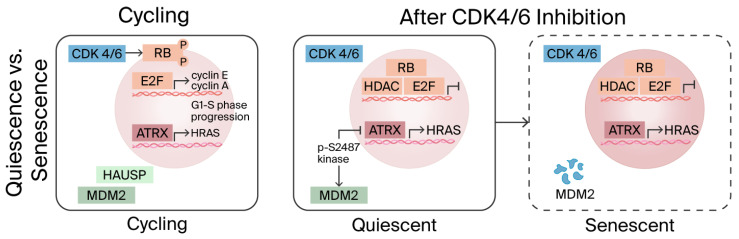
CDK4/6 inhibitors and senescence. Adapted from Klein ME et al. Cancer Cell. 2018 [36]. Figure 4 has been expanded to detail the RB–E2F axis, ATRX-associated chromatin remodeling, the repression of MYC/HRAS, SASP formation, CDK9/MCL1-related transcriptional effects, and potential GSK3β/WNT interactions, with the original conceptual basis referenced from Klein et al.

Figure 4 has been expanded in the text to detail the RB–E2F axis, ATRX-associated chromatin remodeling, the repression of MYC/HRAS, SASP formation, CDK9/MCL1-related transcriptional effects, and potential GSK3β/WNT interactions, with the original conceptual basis appropriately referenced to Klein et al. [36].

(c) Immunomodulation by CDK4/6 inhibitors

Preclinical evidence suggests that CDK4/6 inhibitors exert antitumor effects not only through senescence but also through immunomodulation, which is a second, and perhaps equally critical, component of their therapeutic action. By positioning immune effects as a co-equal driver of the “carry-over” effect, new possibilities for synergy with immune checkpoint inhibitors can be explored [37]. Proposed mechanisms include cell cycle blockades, modulation of energy metabolism, induction of autophagy, and enhancement of immune responses. These effects arise through interactions between tumor cells and immune cells, tumor–antigen presentation, and SASP release that leads to recruitment of immune cells, activation of effector T lymphocytes, depletion of regulatory T cells (Tregs), and depletion of myeloid-derived suppressor cells (MDSCs), which are potent inhibitors within the tumor microenvironment [38].

(d) Biological pathways that may contribute to “carry-over” effect

The “carry-over” effect of CDK4/6 inhibitors is likely not a single phenomenon in HR+/HER2− EBC, but rather the occurrence of multiple events such as eradication of micrometastatic disease during a critical treatment period (preventing the growth of metastatic niches in bone or lymph nodes in HR+/HER2− breast cancer) or cellular reprogramming that leads to non-proliferative states with durable responses (senescence) [39]. In a study conducted in mice and breast cancer patients, CDK4/6 inhibition was observed to reveal a senescent state through SASP activation in p53-enriched targets, without reliance on pro-inflammatory components [40].

Another event is a “post-treatment” effect that persists during follow-up. In NATALEE, the iDFS benefit after completing 3 years of ribociclib reinforces the “carry-over” hypothesis, supporting the idea that a finite duration of a CDK4/6 inhibitor can translate into long-term iDFS gains (while OS remains immature, the trend favors improvement). In this review, the “post-treatment” effect is used descriptively to denote the benefit observed after treatment discontinuation, whereas the “carry-over” effect refers to the broader concept that such sustained post-treatment benefit reflects a persistent therapeutic impact of a finite intervention.

## 4. Future Directions

The following considerations are recommended for future studies:

- Statistical analysis and trial design: Interpreting the “carry-over” effect may require statistical caution, as Kaplan–Meier curves can show delayed separation or non-proportional hazards depending on the treatment, making time-period analyses important. In addition, differences in trial design, high treatment discontinuation rates, and potential informative bias complicate interpretation. Although iDFS is an emerging endpoint in EBC, it has not yet been validated as a surrogate endpoint for OS in this setting [41].
- Type of oncologic treatment and “delayed effects”: Some treatments, such as immunotherapy, can exhibit delayed effects, which are characteristic of these agents. In contrast, targeted therapies, such as CDK4/6 inhibitors, produce faster response times (8–9 weeks) and, with the “carry-over” effect, may also maintain post-treatment responses [42].
- Biomarkers: In HR+/HER2− EBC, several biomarkers of early resistance are being evaluated, such as cyclin E1 (CCNE1) amplification, which may explain failure with palbociclib and ribociclib in certain patient subgroups through parallel activation of the CDK2 pathway, “bypassing” CDK4/6 inhibition and leading to accelerated tumor growth and poor outcomes in the metastatic setting [43].
- Monitoring of minimal residual disease (MRD): Another emerging biomarker is circulating tumor DNA (ctDNA), which can detect MRD and guide strategies for treatment intensification or de-escalation [44]. Currently, the implementation of CCNE1 amplification or ctDNA MRD testing in routine clinical practice varies. While some centers have started incorporating these tests to better stratify patient treatment plans, their broader use remains limited due to the need for further validation and consensus on clinical guidelines. Clinicians should consider the availability of these biomarkers and integrate them into treatment discussions where applicable to tailor therapies more effectively [45].
- Treatment exposure and adherence: The clinical relevance of the “carry-over” effect depends on patients achieving sufficient drug exposure. Therefore, the prevention and proactive management of toxicities (e.g., diarrhea with abemaciclib, transaminitis and QTc prolongation with ribociclib), as well as maintaining adherence, are essential to preserving the “carry-over” mechanism. Since these patients receive treatment in a non-metastatic setting, it is crucial to emphasize that, in HR+/HER2− EBC, adjuvant therapy is not only about efficacy but also about quality of life and tolerability, including discontinuation due to adverse events from adjuvant CDK4/6 inhibitors [46].
- Since adjuvant CDK4/6 inhibition is administered for a limited period, toxicity management is not merely a matter of supportive care but a key determinant of biologically meaningful treatment exposure. A potential “carry-over” effect can only be observed if patients remain on treatment long enough and at sufficient dose intensity to modify micrometastatic disease. Consequently, structured management of diarrhea with abemaciclib and protocol-based monitoring of neutropenia, liver function tests, and the QTc interval with ribociclib should be considered integral components of treatment efficacy, rather than merely adjunctive measures [47].
- “Financial toxicity”: The decision of agencies such as the National Institute for Health and Care Excellence (NICE) to approve the use of CDK4/6 inhibitors, such as abemaciclib and ribociclib, highlights the relevance of the “carry-over” effect as a treatment consideration that impacts health system budgets (treatment duration + cost + adherence + toxicity and toxicity-related costs) [48,49].
- “Replacement” strategies in HR+/HER2− EBC: Recently, at the 2025 San Antonio Breast Cancer Symposium (SABCS), the lidERA study showed an iDFS benefit with 30 mg of giredestrant [a next-generation oral selective estrogen receptor degrader (SERD)] orally once daily (QD) vs. ET alone (3-year iDFS: 92.4% with giredestrant vs. 89.6% with standard ET; HR 0.70; 95% CI 0.57–0.87; *p* = 0.014) [50]. This strategy of replacing classic ET (tamoxifen and NSAI) with an oral SERD is likely to generate intense debate between intensification vs. replacement strategies for adjuvant therapy in HR+/HER2− EBC. Currently, giredestrant and similar SERDs are being reviewed for regulatory approval, with expectations that they will be recommended for HR+/HER2− EBC patients who may not benefit optimally from traditional ET. These advancements could significantly influence clinical decision-making by offering a tailored approach to treatment.

## 5. Conclusions

Current evidence supports durable iDFS benefit with abemaciclib and ribociclib in selected HR+/HER2− EBC populations; however, only abemaciclib has thus far shown a statistically significant, albeit modest, emerging OS benefit, whereas ribociclib OS remains immature. Therefore, future progress will depend less on broad claims of drug-specific superiority and more on improving patient selection, treatment adherence, and toxicity management.

The magnitude and duration of the “carry-over” effect depend not only on the drug itself, but also on other factors that may influence outcomes, such as trial design (sample size, “high-risk” criteria, drug duration or dose, and discontinuation rate, among others). Therefore, the “carry-over” effect is a multistage clinical–molecular “phenomenon” that also depends on treatment adherence.

In clinical practice, “carry-over” is clinically relevant when it is realized through adherence to therapy, including strategies to manage toxicities and sustain adherence. Treatment discontinuation can diminish the potential post-treatment benefit.

Available studies support the use of abemaciclib and ribociclib, which provide iDFS benefit (and abemaciclib has also demonstrated an OS improvement), reinforcing the concept that a finite adjuvant intervention with a CDK4/6 inhibitor translates into a durable impact in a potentially curative setting. The lack of benefit with palbociclib, contrasted with favorable results in some biomarker-defined subgroups, suggests that CDK4/6 inhibitor selectivity is determinant, and use may be considered in specific clinical–molecular profiles.

Evaluating potential biomarkers (TKa, pRB, MRD via ctDNA, etc.) will enable identification of EBC populations at risk for early relapses and help to define treatment intensification or de-escalation strategies, optimizing benefit while reducing toxicity. Emerging therapies such as adjuvant oral SERDs (giredestrant) broaden the debate: intensification (CDK4/6 inhibitor + ET) will likely be used in high-risk patients, while replacement of ET with SERDs may be used in selected subgroups. “Carry-over” may be a key criterion to guide the best treatment strategy. However, a critical question remains: which patients should we de-escalate first? Researchers and clinicians should translate insights from the “carry-over” effect into the development of new trial concepts, ultimately aiming to refine patient selection and enhance therapeutic outcomes.

The implications of “carry-over” extend beyond clinical efficacy; “financial toxicity” and health system sustainability are also relevant, especially for innovative, high-cost treatments administered for years. These factors position the “carry-over” effect as a valid concern to justify (or question) treatments in the context of health policy.

## Data Availability

The original contributions presented in this study are included in the article. Further inquiries can be directed to the corresponding author.

## Abbreviations

The following abbreviations are used in this manuscript:

Δ	Absolute difference
AE	Adverse event
ATP	Adenosine triphosphate
ATRX	Alpha-thalassemia mental retardation X-linked
BID	Twice per day
CCNE1	Cyclin E1 amplification
CDK 4/6	Cyclin-dependent kinase 4/6
CPS-EG	Combining clinical/pathological stage and estrogen receptor status/grade
ctDNA	Circulating tumor DNA
DRFS	Distant recurrence-free survival
EBC	Early breast cancer
ER	Estrogen receptor
erbB2	HER2/neu
ET	Endocrine therapy
GR50	Growth rate 50 inhibition
GSK3β	Glycogen synthase kinase 3 beta
HR+	Hormone-receptor-positive
IC50	Half-maximal inhibitory concentration
iDFS	Invasive disease-free survival
LBC	Lobular breast cancer
MCL-1	Myeloid cell leukemia-1
MDSC	Myeloid-derived suppressor cell
MRD	Minimal residual disease
nM	Nanomolar
NICE	National Institute for Health and Care Excellence
NSAI	Nonsteroidal aromatase inhibitor
OS	Overall survival
PgR	Progesterone receptor
PK	Pharmacokinetics
PPI	Proton pump inhibitor
pRB	Retinoblastoma protein
P-TEFb	Positive transcription elongation factor b
QTc	Corrected Q-T interval
SABCS	San Antonio Breast Cancer Symposium
SASP	Senescence-associated secretory phenotype
SERD	Selective estrogen receptor degrader
TKa	Thymidine kinase 1
Treg	Regulatory T cell
ypN+	Positive node involvement at surgery
